# Sequence analyses at mitochondrial and nuclear loci reveal a novel *Theileria* sp. and aid in the phylogenetic resolution of piroplasms from Australian marsupials and ticks

**DOI:** 10.1371/journal.pone.0225822

**Published:** 2019-12-18

**Authors:** Amanda D. Barbosa, Jill Austen, Timothy J. Portas, J. Anthony Friend, Liisa A. Ahlstrom, Charlotte L. Oskam, Una M. Ryan, Peter J. Irwin

**Affiliations:** 1 Vector- and Water-Borne Pathogen Research Group, College of Science, Health, Engineering and Education, Murdoch University, Murdoch, WA, Australia; 2 CAPES Foundation, Ministry of Education of Brazil, Brasília—DF, Brazil; 3 Veterinary and Research Centre, Tidbinbilla Nature Reserve, Australian Capital Territory, Australia; 4 Department of Biodiversity, Conservation and Attractions, Albany, WA, Australia; 5 Bayer Australia Ltd, Pymble, Australia; Institut national de la santé et de la recherche médicale - Institut Cochin, FRANCE

## Abstract

The order Piroplasmida encompasses two main families: *Babesiidae* and *Theileriidae*, containing tick-borne pathogens of veterinary and medical importance worldwide. While only three genera (*Babesia*, *Cytauxzoon* and *Theileria*) comprising piroplasm parasites are currently recognised, phylogenetic studies at the 18S rRNA (18S) gene suggest that these organisms represent at least ten lineages, one of which comprises the relatively unique and highly diverse *Theileria* spp. from Australian marsupials and ticks. As an alternative to analysing 18S sequences alone, sequencing of mitochondrial genes has proven to be useful for the elucidation of evolutionary relationships amongst some groups of piroplasms. This research aimed to characterise piroplasms from Australian native mammals and ticks using multiple genetic markers (18S, cytochrome c, oxidase subunit III (*cox3)* and cytochrome B (c*ytB*)) and microscopy. For this, nearly complete piroplasm-18S sequences were obtained from 32 animals belonging to six marsupial species: eastern bettong (*Bettongia gaimardi*), eastern quoll (*Dasyurus viverrinus*), eastern grey kangaroo (*Macropus giganteus*), swamp wallaby (*Wallabia bicolor*), quokka (*Setonix brachyurus*) and Gilbert’s potoroo (*Potorous gilbertii*). The organisms detected represented eight novel *Theileria* genotypes, which formed five sub-clades within the main marsupial clade containing previously reported Australian marsupial and tick-derived *Theileria* spp. A selection of both novel and previously described Australian piroplasms at the 18S were also successfully characterised, for the first time, at the *cox3* and *cytB* loci, and corroborated the position of Australian native theilerias in a separate, well-supported clade. Analyses of the *cox3* and *cytB* genes also aided in the taxonomic resolution within the clade of Australian Piroplasmida. Importantly, microscopy and molecular analysis at multiple loci led to the discovery of a unique piroplasm species that clustered with the Australian marsupial theilerias, for which we propose the name *Theileria lupei* n. sp.

## Introduction

Piroplasm is a generic term used to describe apicomplexan haemoprotozoan parasites belonging to the order Piroplasmida. This order encompasses two main families: *Babesiidae* and *Theileriidae*, containing three main genera (*Babesia*, *Theileria* and *Cytauxzoon*) which are tick-borne pathogens of veterinary and medical importance worldwide [1, 2]. Piroplasms appear to be ubiquitous in Australia, with 24 unique species and genotypes described to date in wildlife and ticks, often at a high prevalence within the unique native fauna [3–7]. However, relatively little is still known about the clinical significance and zoonotic potential of these Australian parasites [5].

Classical taxonomy of piroplasms has been based predominantly on transmission dynamics, host cell type(s) infected, vertebrate host preferences, and parasite morphology [8, 9]. *Theileria* and *Cytauxzoon* species are thought to be limited to transstadial transmission in the tick and undergo extra-erythrocytic schizogony in nucleated cells, prior to the erythrocytic stage. Although *Cytauxzoon* was originally established as a distinct genus from *Theileria* due to its invasion of monocytes rather than lymphocytes [10], species in both genera were later shown to infect both lymphocytes and monocytes [11]. Piroplasms belonging to the *Babesia* sensu stricto taxonomic group exhibit transstadial and transovarial transmission in the tick and exclusive infection of erythrocytes in the vertebrate host. *Babesia* spp. that either undergo schizogony in the vertebrate host or lack transovarial transmission in the tick host have been referred to as *Babesia* sensu lato [12]. In addition, based on morphological features, the babesias are broadly grouped into two forms: small *Babesia* (trophozoites are 1.0 to 2.5 μm; e.g., *B*. *gibsoni*, *B*. *rodhaini*) and large *Babesia* (2.5 to 5.0 μm; e.g., *B*. *bovis*, *B*. *caballi*) [13].

As more molecular and biological information has been discovered, it has become evident that this classification system is limited and fails to reflect the diversity and evolution of the Piroplasmida [8, 14, 15]. While only three main genera which comprise piroplasms of medical and veterinary importance are formally recognised, previous phylogenetic studies at the 18S rRNA gene (hereafter referred to as 18S) have demonstrated that these organisms represent a polyphyletic assemblage comprising at least six principal lineages [1, 8, 15–18]. Of these, two lineages were first revealed by recent phylogenetic studies based on nearly full-length 18S sequences from *Theileria* spp. isolated from Australian native marsupials, monotremes and ticks [6, 7, 19–21].

As an alternative to analysing 18S sequences alone, sequencing of non-nuclear mitochondrial genes has proven to be useful for elucidation of evolutionary relationships and for delineating specimens to the species level for a number of eukaryotes. More recently, this molecular strategy was successfully applied to a few groups of piroplasms and has provided corroborative evidence that these organisms may in fact represent more than only three genera of parasites [8].

In the light of these important taxonomic challenges, there is a need for current research to further investigate the diversity and systematics of piroplasmid parasites, including those presumably unique to Australia. For this, further taxon sampling and the synthesis of more comprehensive genetic data (at various loci) are crucial. In addition, an integrative taxonomic approach involving biogeography, morphology and ecology is also essential for a more reliable taxonomic classification of these organisms in the future [22]. In this context, the aims of this research were to: (1) identify and/or further characterise piroplasms from Australian native mammals and ticks based on nearly full-length 18S, cytochrome c oxidase subunit III (*cox3)* and cytochrome B (c*ytB*) sequences; (2) identify and morphologically characterise intra-erythrocytic inclusions and schizonts in white blood cells (a biological trait of the true theilerias) in blood films from Australian marsupials; and (3) further investigate the host-range and spatial distribution of Australian native piroplasms.

## Materials and methods

### Sampling

This study comprised a total of 45 marsupial blood samples and ticks from various locations across Australia. Of these, six blood samples were collected from animals belonging to four marsupial species housed at Tidbinbilla Nature Reserve in the Australian Capital Territory (ACT): eastern bettong (*Bettongia gaimardi*), eastern quoll (*Dasyurus viverrinus*), eastern grey kangaroo (*Macropus giganteus*) and swamp wallaby (*Wallabia bicolor*) (Table 1). Eastern bettongs and quolls were anaesthetised using isoflurane in oxygen delivered via mask and blood was collected from the jugular vein. Eastern grey kangaroos and swamp wallabies were anaesthetised using a proprietary combination of tiletamine (5 mg/kg) and zolazepam (5mg/kg) delivered intramuscularly via a projectile syringe and blood was collected from the recurrent tarsal vein. All blood samples were added to commercial blood storage tubes containing ethylene diamine tetra-acetic acid (EDTA) (Sarstedt, Australia) and stored at -20°C until processed at Murdoch University. An additional 24 blood samples sourced from quokkas (*Setonix brachyurus*) from Two Peoples Bay and Bald Island in Western Australia (WA) [23], were tested for piroplasms for the first time in the present study (Table 1). The sampling was conducted using standard procedures approved by the Murdoch University Animal Ethics Committee (Permits W2204/09 and RW2636/14), Department of Parks and Wildlife (Permit number SC000767) and DPAW Animal Ethics Committee approval 36–2008.

**Table 1:** Blood samples collected from Australian marsupials screened for piroplasms in this study.

Sample code	Host species	Location
TP8	Eastern bettong (*Bettongia gaimardi*)	Tidbinbilla Reserve, ACT
TB27		
TP13	Eastern grey kangaroo (*Macropus giganteus*)	
TB102	Eastern quoll (*Dasyurus viverrinus*)	
TB105		
TB111	Swamp wallaby (*Wallabia bicolor*)	
Q1051, Q1837, Q4137, Q1349, Q1340, Q1362, Q4166, Q1368, Q2031a, Q2031b, Q1355, Q1356, Q1407, Q1404, Q1339, Q2324, Q1416, Q2399	Quokka (*Setonix brachyurus*)	Two Peoples Bay, WA
Q2522, Q1947, Q24, Q3547, Q2367, Q2377		Bald Island, WA

WA: Western Australia; ACT: Australian Capital Territory

A further 15 DNA samples from Australian marsupials’ blood and ticks, known to be positive for piroplasms (Table 2) [3, 6, 7, 20] were also investigated. Importantly, eight of these samples consisted of the type-specimens formally attached to the species: *T*. *gilberti*, *T*. *paparinii*, *T*. *apogeana*, *T*. *palmeri*, *T*. *worthingtonorum*, *B*. *lohae* and *B*. *mackerrasorum* [3, 7].

**Table 2:** Samples positive for piroplasm 18S rDNA from wildlife and ticks utilised in this study.

Sample code	Specimen	Vertebrate host	Location	Reference study	Piroplasm detected	18S GenBank Accession Number	Approximate18S rDNA fragment length
P68	Blood	Gilbert’s potoroo (*Potorous gilbertii*)	Two Peoples Bay, WA	Lee et al., 2009	*T*. gilberti*	EF554394	600bp
P92	Blood	Gilbert’s potoroo	Two Peoples Bay, WA	Lee et al., 2009	*T*. gilberti*	EF554395	600bp
ITF4	*Ixodes tasmani*	Dog	Lower Wilmot, Tas.	Greay et al., 2018	*T*. paparinii*	MG758115	1.5kb
ITF7	*I*. *tasmani*	Dog	Devonport, Tas.	Greay et al., 2018	*T*. apogeana*	MG758116	1.5kb
ITF6	*I*. *tasmani*	Dog	Port Sorell, Tas.	Greay et al., 2018	*T*. palmeri*	MG758113	1.5kb
ITF3	*I*. *tasmani*	Dog	Port Sorell, Tas.	Greay et al., 2018	*T*. worthingtonorum*	MG758114	1.5kb
IHF1	*I*. *holocyclus*	Cat	Park Ridge, Qld	Greay et al., 2018	*B*. lohae*	MG593272	1.5kb
HspM1	cf. *Haemaphysalis*	Horse	Tanja, New South Wales	Greay et al., 2018	*B*. mackerrasorum*	MG593271	1.5kb
BP1	*I*. *tasmani*	Brushtail possum (*Trichosurus vulpecula*)	Royal Botanic Garden, NSW	Loh et al., 2018	*Babesia sp*.	MG251435	1.3kb
BP7	*I*. *tasmani*	Brushtail possum	Beerwah, Qld	Loh et al., 2018	*Babesia sp*.	MG251436	1.3kb
B16	*I*. *tasmani*	Bandicoot sp.	Port Sorel, Tas.	Loh et al., 2018	*Theileria sp*.	MG251437	1.3kb
B43	*I*. *tasmani*	Long-nosed bandicoot (*Peramelis nasuta*)	Beerwah, Qld	Loh et al., 2018	*Theileria sp*.	MG251438	1.3kb
B60	*I*. *tasmani*	Long-nosed bandicoots	Beerwah, Qld	Loh et al., 2018	*Theileria sp*.	MG251439	1.3kb
K1	Blood	Boodie (*Bettongia lesueur*)	Kanyana, WA	Paparini et al., 2012	*Theileria sp*. isolate K1	JQ682879	1.4kb
K36	*I*. *australiensis*	Western Grey Kangaroo (*Macropus fuliginosus*)	Lowlands, WA	Loh et al., 2018	*Theileria* sp.	MF576261	1.3kb

* indicates type-specimens / WA: Western Australia; NSW: New South Wales; Qld: Queensland; Tas.: Tasman

### DNA extraction

Genomic DNA was isolated from 200 μl of whole blood in EDTA using the MasterPure Purification Kit (Epicentre Biotechnologies, USA), according to the manufacturer’s instructions. A mock extraction from reagents and fresh consumables was included in each DNA extraction to exclude possible contaminations with piroplasm DNA (extraction reagent blank).

### 18S rRNA and mitochondrial genes amplification and sequencing

New samples (i.e. those which had not yet been tested for piroplasms) (Table 1) or from which only relatively short (~600–800 bp) piroplasm 18S sequences were available to date were screened using a single-round PCR (with primers Nbab_1F and 18SApiR), previously designed to amplify an approximately 1.4 kb piroplasm 18S fragment [7]. In addition, due to the fact that a different genotype was amplified from two *T*. *gilberti* type-specimens, the nested PCR originally used by Lee *et al*. [3], targeting an approximately 800 bp fragment [24], was performed to confirm the hypothesis of mixed infections in those samples. This same alternative method was used to screen quokka blood samples, from which a novel genotype was isolated using the protocol established by Greay et al. [7]. The aim of the additional testing was to investigate if *T*. *brachyuri* (previously described in quokkas) was concurrently present in the samples.

Subsequently, fragments of the *cox3* (~600 bp) and *cytB* (~1 kb) genes were amplified from samples collected in the present study from which unique 18S sequences, representative of each clade revealed by phylogenetic analysis, were obtained. Additionally, all samples positive for piroplasms previously described at the 18S locus (Table 2), were tested for the first time at both *cox3* and *cytB* loci. A modified protocol from Schreeg *et al*. [8] was performed, in which each 50 μL PCR reaction contained 4 μL of DNA template, 1× KAPA Taq buffer (Sigma-Aldrich, St. Louis, Missouri, USA), 2 mM MgCl_2_, 1 mM dNTPs, 400 nM of each primer, and 0.02 U KAPA Taq DNA Polymerase (Sigma-Aldrich). Thermal cycling conditions consisted of an initial denaturation at 94°C for 5 minutes, followed by 50 amplification cycles including 94°C for 20 seconds, 55°C (*cox3*) or 56°C (*cytB*) for 30 seconds, and 68°C for 45 seconds (*cox3*) or 1 minute (*cytB*). A final extension step was performed at 72°C for 7 minutes. DNA extraction blank controls, no-template controls (2 μL of ultra-pure water) and positive control (*Babesia gibsoni* DNA) were included in all assays.

PCR products were run on a 2% agarose gel stained with SYBR Safe Gel Stain (Invitrogen, USA) and visualised with a dark reader trans-illuminator (Clare Chemical Research, USA). Amplicons corresponding to the expected length were excised from the gel, purified using an in-house filter tip method [25] and sequenced in both directions using an ABI Prism Terminator Cycle Sequencing kit (Applied Biosystems, USA), on an Applied Biosystem 3730 DNA Analyser. Unique DNA sequences (i.e. duplicates removed) obtained during the present study are available in GenBank (https://www.ncbi.nlm.nih.gov/genbank/) under the following accession numbers: KU641429, MN101137-MN101145, and MN135557- MN135576.

### Phylogenetic analyses

Forward and reverse sequence chromatograms obtained in this study were aligned and merged to generate consensus sequences using Geneious v10.0.9 (https://www.geneious.com). For each locus (18S, *cox3* and *cytB*), Neighbour-Joining (NJ) and Maximum Likelihood (ML) methods were performed on the consensus sequences and additional sequences from a selection of piroplasm taxa retrieved from GenBank. This selection included representatives of the major resolved clades within the order Piroplasmida [1, 8] and sequences from piroplasms previously recorded in Australian mammals and ticks [3, 6, 7, 19–21, 26–29]. Two separate phylogenetic analyses were conducted at the 18S locus, one containing only nearly complete or full length sequences, and the other also containing partial sequences from Australian piroplasms. In both cases, all sequences were trimmed to the length of the shortest sequence in the alignment.

In Geneious v10.0.9, multiple 18S sequence alignment was performed by the MAFFT v7 plugin [30] under the default settings, prior to global trimming and complete gap removal using the function: “tools → mask alignment → sites containing any gaps”. Bootstrapped (n = 500 replicates) NJ and ML phylogenies were constructed after testing in MEGA 7 [31] for nucleotide substitution models with the lowest Bayesian information criterion (BIC) scores. The models selected for analyses of the longer and shorter 18S alignments were the General Time Reversible (GTR) [32] and the Tamura-Nei model (TN) [33], respectively. A discrete Gamma distribution was used to model evolutionary rate differences among sites (5 categories, +G, parameter = 0.1892 and 0.1475, respectively).

Alignments of *cox3* and *cytB* sequences were also produced using MAFFT v7 [30] in Geneious, and then imported into MEGA 7 under the options “import protein-coding -> protozoan mitochondrial sequences”. The models with lowest BIC scores selected for *cox3* and *cytB* sequence alignments were the GTR and TN, respectively. All trees were drawn to scale, with branch lengths measured in the number of substitutions per site. Accession numbers of sequences retrieved from GenBank used in the analyses are shown in parentheses. Bootstrap values (>60%) are indicated at the left of each node.

Estimates of genetic distances between 18S sequences were calculated in MEGA 7 using the GTR and TN models for the longer and shorter alignments, respectively. The rate variation among sites was modelled with a gamma distribution (shape parameter = 0.16 and 0.14, respectively). Genetic distances between *cox3* and *CytB* sequences were computed using the p-distance method, also in MEGA 7. For simplicity, duplicates were excluded from the sequence databases prior to the genetic distances analyses.

### Microscopy analysis

Thin blood smears were made from whole blood, prepared by smearing a drop of blood onto a microscope slide, which was then air dried and stained with Modified Wright stain, using an automated slide stainer (Ames Hema-Tek®, Bayer, Germany). A cover-slip was placed over the stained blood smear and the preparation was examined microscopically at 200× and 400× magnification to check for the presence of piroplasms. Smears were then observed at 1000× magnification and images of the parasites recorded using an Olympus DP71 Advance digital camera. The parasites’ dimensions were measured using the software Image J [34].

### Nomenclatural acts

The electronic edition of this article conforms to the requirements of the amended International Code of Zoological Nomenclature, and hence the new names contained herein are available under that Code from the electronic edition of this article. This published work and the nomenclatural acts it contains have been registered in ZooBank, the online registration system for the ICZN. The ZooBank LSIDs (Life Science Identifiers) can be resolved and the associated information viewed through any standard web browser by appending the LSID to the prefix “http://zoobank.org/”. The LSID for this publication is: urn:lsid:zoobank.org:pub:F7C6784A-E4F0-4392-9720-468BFC030BD2. The electronic edition of this work was published in a journal with an ISSN, and has been archived and is available from the following digital repositories: PubMed Central, LOCKSS [author to insert any additional repositories].

## Results

### Molecular characterisation and phylogeny of Australian piroplasms at the 18S rRNA locus

**Fig 1:**
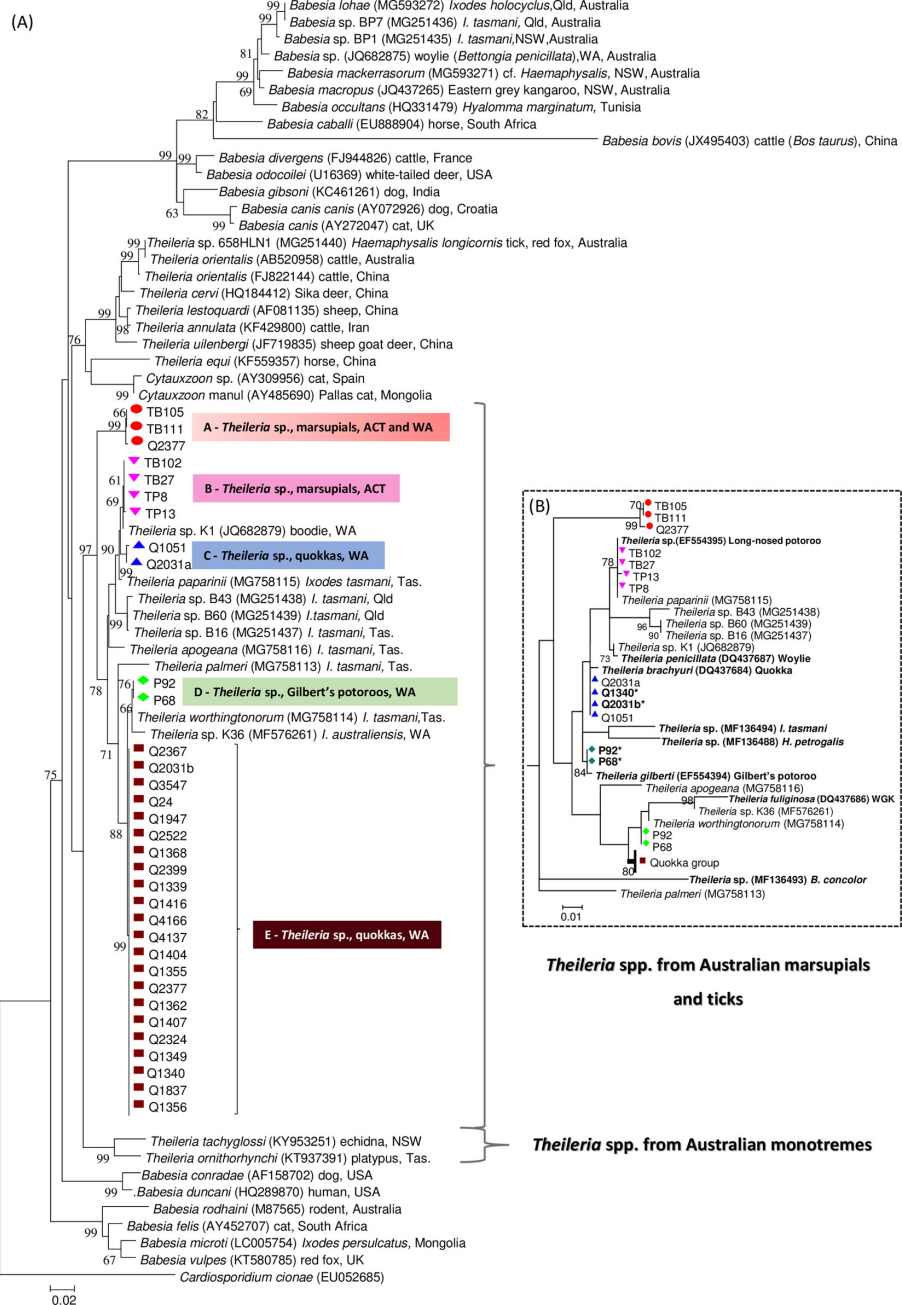
Phylogenetic analysis of newly reported *Theileria* spp. and other piroplasms based on the 18S rRNA locus. (A) Phylogenetic relationships among piroplasm 18S rDNA sequences (~ 1.3 kb) were inferred using the maximum likelihood method. (B) Maximum likelihood analysis of piroplasms based on a 800bp alignment to illustrate the position of newly reported organisms in the context of all known *Theileria* spp. recorded in Australia to date. Names in bold represent sequences retrieved from GenBank that were not included in the main tree (Fig 1, A) due to their shorter length. GenBank accession codes are shown in parentheses. Bootstrap values (>60%) based on 500 replicates are indicated at the left of each node. * indicates shorter sequences (~ 800 bp) obtained in this study using a nested PCR as described by Jefferies et. al. (2007). All other new sequences (~1.3 kb) were obtained using primers Nbab_1F and 18S ApiR (Greay et al., 2018).

All of the 33 marsupial blood samples tested by PCR in the present study generated piroplasm 18S sequences. Both NJ (data not shown) and ML analyses of newly reported and previously described 18S sequences yielded concordant tree topologies, and confirmed the polyphyletic character of the order Piroplasmida. In the present phylogenetic reconstruction, members of the Australian marsupial/tick clade were distinct from their overseas counterparts and grouped neither with the true theilerids nor with the more ancestral *T*. *ornithorhynchi/ T*. *tachyglossi* group. Furthermore, the 33 nearly complete marsupial-derived piroplasm 18S sequences obtained in the present study represented eight distinct *Theileria* genotypes, which formed five sub-clades (A-E) within the main clade containing previously reported Australian marsupial/tick-derived *Theileria* (Fig 1A).

The novel 18S sequences obtained from one eastern quoll (TB105) and a swamp wallaby (TB111) were identical to each other, whereas a novel genotype isolated from a quokka (Q2377) contained three single nucleotide polymorphisms (SNPs) compared to TB105 and TB111. All three genotypes described above grouped together and formed a separate clade (A) with strong statistical support (99%), positioned within the Australian piroplasms’ main clade (Fig 1A). Clade A is distinct but most similar to *Theileria* sp. K1 from a burrowing bettong or “boodie” (*Bettongia lesueur*), which belongs to the *T*. *paparinii* clade, with a relatively large genetic distance of 2.8%. The fact that this distance at the 18S locus is significantly greater than the minimum distance (0.3%) between two previously named piroplasm species (*T*. *annulata and T*. *lestoquardi*), and pairwise distances between many other known piroplasm species (e.g. *T*. *apogeana* vs *T*. *paparinii*—GD = 1.9%) (S1 Table) indicates these newly reported marsupial-derived *Theileria* genotypes may represent a novel species. Phylogenetic reconstruction and genetic distances based on a shorter alignment (~600 bp) (Fig 1; S1 and S2 Tables) confirm the novel clade of *Theileria* spp. is indeed unique and differs from all other known piroplasms recorded in Australia to date, including *T*. *gilberti*, *T*. *brachyuri*, *T*. *fuliginosa* and *T*. *penicillata*, for which only relatively shorter sequences were available.

Additional four piroplasm 18S sequences were amplified from an eastern quoll (TB102), two eastern bettongs (TB27 and TP8) and an eastern grey kangaroo (TP13). These sequences were identical to each other, except for TP13 (one SNP), and formed a subclade (denoted as clade “B” in Fig 1A) within the *T*. *paparinii* clade. Genetic distances ranging from 0.4% to 0.5% at the 18S were observed between these new genotypes and *T*. *paparinii*, suggesting they may potentially represent a distinct species. Phylogenetic analysis based on a shorter alignment (Fig 1B) confirms the phylogenetic position of these newly reported genotypes and shows that the same *Theileria* sp. detected in samples TB102, TB27 and TP8 have previously been reported in a long-nosed potoroo (*Potorous tridactylus*) from Victoria [3].

Two novel *Theileria* genotypes obtained from quokkas (Q1051 and Q2031a) clustered together in a unique clade supported by a high bootstrap value (99%). The clade containing these two quokka-derived piroplasms was also positioned within the *T*. *paparinii* clade. However, estimates of evolutionary distances of 0.8% to 1.0% observed between the new genotypes and *T*. *paparinii* suggested it may classify a distinct species (S1 Table). Furthermore, phylogenetic analysis and corresponding pairwise distances matrix containing all Australian piroplasms (Fig 1B and S2 Table, respectively) revealed that sequences isolated from Q2031a and Q1051 exhibited a relatively low genetic distance from *T*. *brachyuri* (0.2%). Collectively, these findings suggests the new quokka-derived genotypes may represent a nearly complete 18S fragment of *T*. *brachyuri*.

Another two novel subclades (D and E) (Fig 1A) were revealed by phylogenetic analyses, which comprised *Theileria* 18S sequences from Gilbert’s potoroos and quokkas, respectively. A genetic distance of only 0.1% was observed between representatives of subclade D and *T*. *worthingtonorum*, suggesting these organisms may belong to the same species. However, because P68 and P92 are type-specimens associated with the previously described *T*. *gilberti*, these samples were re-tested using the original assay performed by Lee et al.[3], with the aim to clarify the unexpected amplification of potential *T*. *worthingtonorum* genotypes from these samples. The new molecular analyses produced sequences that consisted of genetic variants of *T*. *gilberti*, confirming that these samples were co-infected with *T*. *gilberti* and a novel genotype (clade D).

Estimates of genetic distances based on the longer alignment revealed a relatively larger genetic distance (0.5%) between genotypes belonging to clade E and *T*. *worthingtonorum*, suggesting these could potentially represent a unique species. In addition, two of the quokka-derived sequences positive for novel *Theileria* genotypes (Q1340 and Q2031b) using the single-round PCR [7] also generated *T*. *brachyuri* sequences when re-tested using primers sourced from Jefferies et al. [24] (Fig 1B).

### Molecular characterisation and phylogeny of Australian piroplasms at the *cox 3* and *cytB* genes

**Fig 2:**
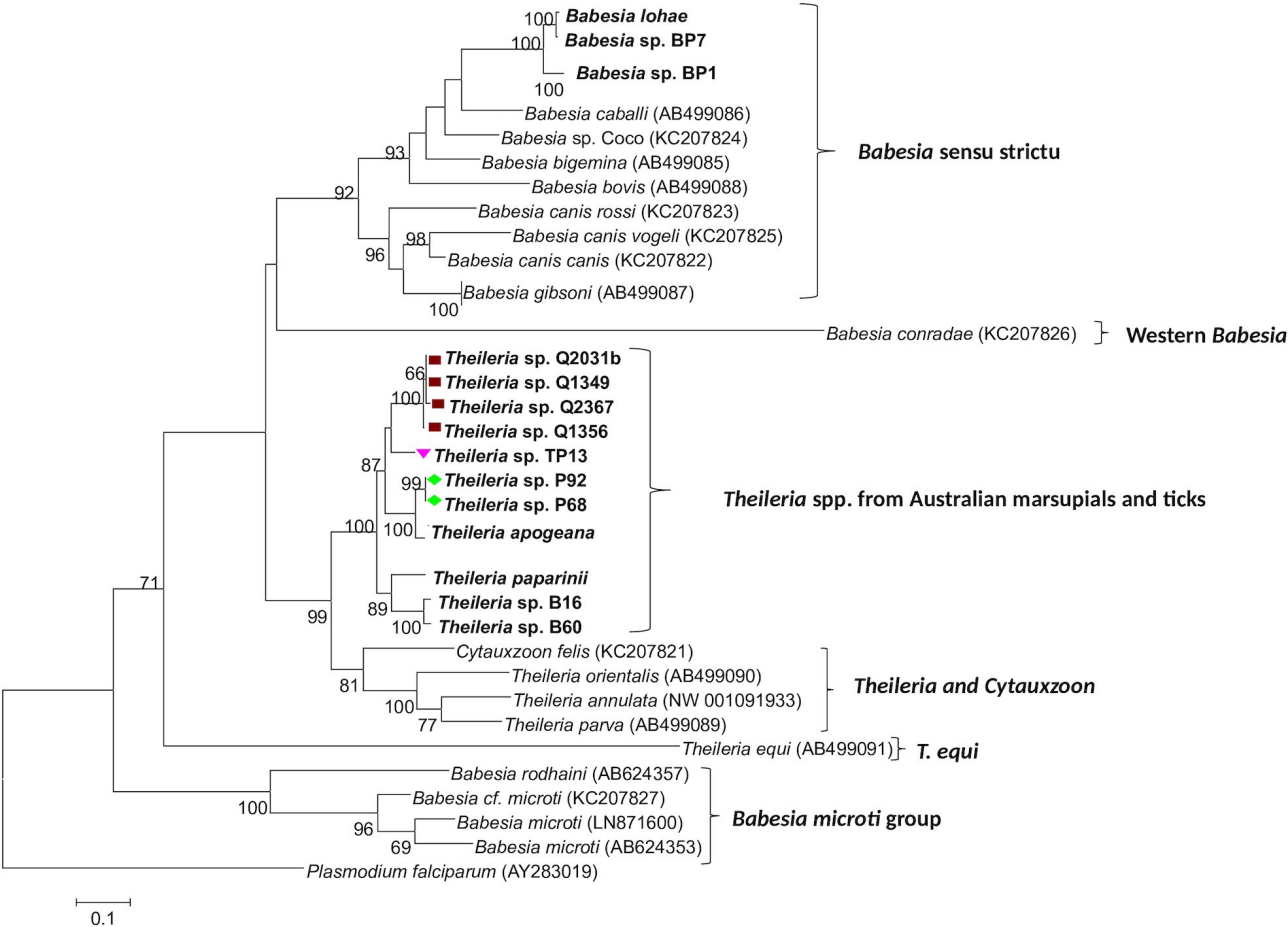
Phylogenetic analysis of *cox3* nucleotide sequences from Australian piroplasms and other piroplasms for which *cox3* sequences are available in GenBank. Phylogenetic relationships among sequences (~ 600 bp) were inferred using the maximum likelihood method. Names in bold represent sequences obtained in the present study from marsupial blood samples (Table 1) and positive piroplasm 18S rDNA samples obtained during previous studies (Table 2). Symbols at the left of each sequence correspond to the clades identified in the phylogenetic analysis at the 18S rRNA locus (Fig 1).

**Fig 3:**
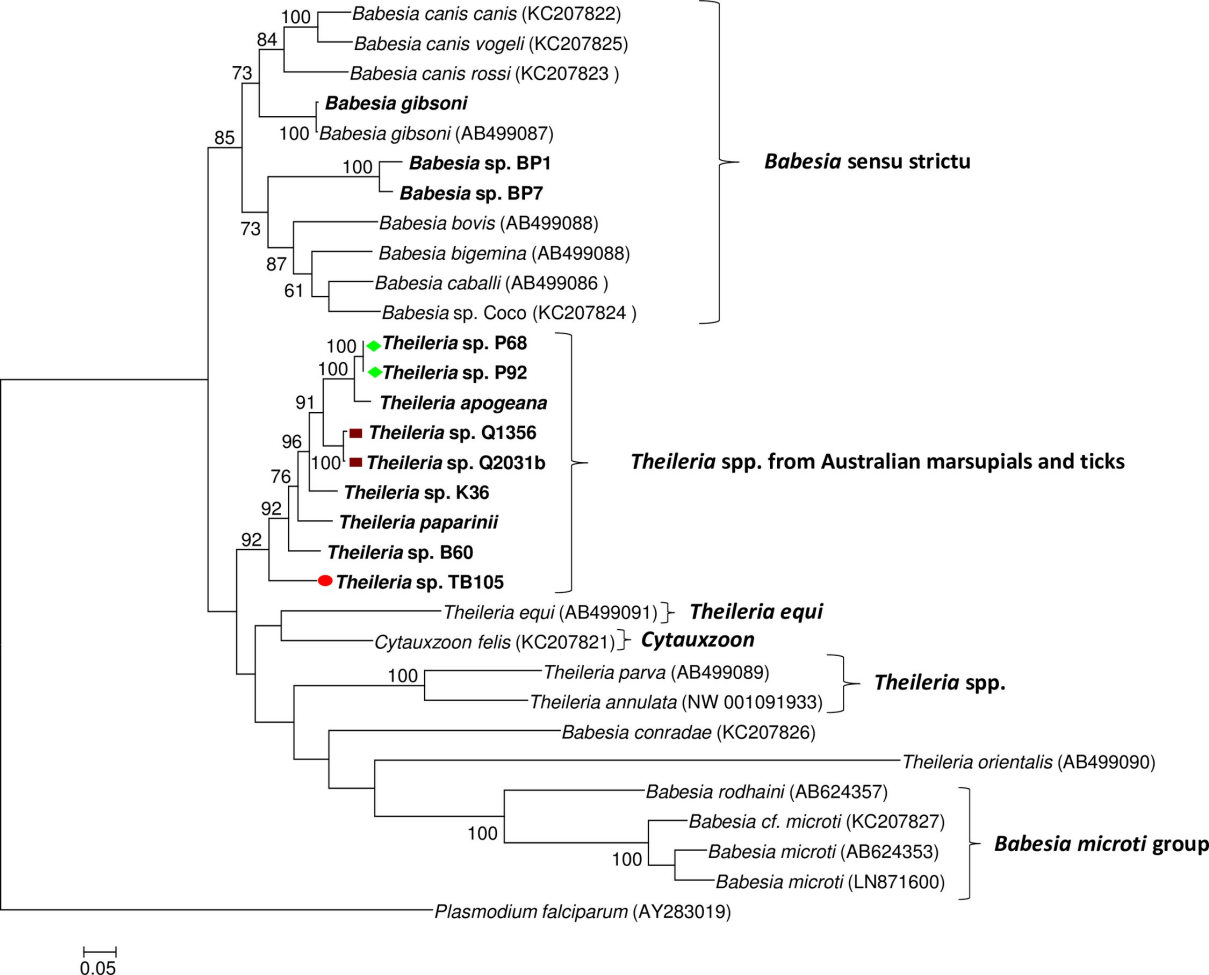
Phylogenetic analysis of *cytB* nucleotide sequences from Australian piroplasms and other piroplasms for which *cytB* sequences are available in GenBank. Phylogenetic relationships among sequences (~1.1 kb) were inferred using the maximum likelihood method. Names in bold represent sequences obtained in the present study from marsupial blood samples (Table 1) and positive piroplasm 18S rDNA samples obtained during previous studies (Table 2). Symbols at the left of each sequence correspond to the clades identified in the phylogenetic analysis at the 18S rRNA locus (Fig 1).

A total of 14 *cox 3* and 12 *cytB* DNA sequences (out of 28 samples tested at both loci) were successfully amplified using molecular assays performed to amplify, for the first time, mitochondrial genes from Australian piroplasms. The evolutionary relationships of the novel sequences amongst each other and with their overseas counterparts (for which genetic information were available at the same loci) are presented in Figs 2 and 3, respectively.

Both NJ (data not shown) and ML analyses at the *cox 3* gene produced similar tree topologies and corroborate the position of Australian native theilerias in a separate, well-supported clade (Fig 2). Interestingly, this analysis revealed a clear paraphyletic relationship between the Australian group and the clade containing *Cytauxzoon* and the true theilerias. In addition, phylogenetic relationships between Australian piroplasms’ *cox3* sequences appear to be better resolved, with most clades well-supported by bootstrap values and less ambiguities compared to the 18S analysis.

The clade containing the Australian marsupial putative *Theileria* spp. (Fig 2) shared approximately 69–74% pairwise identity with their most closely related taxa (i.e. *Theileria* sensu stricto and *Cytauxzoon* spp.), at the *cox 3* locus. Intra-specific and inter-specific genetic distances within this group, on the other hand, ranged from 0.6% (between Q2031 and Q1356; Q1349 and Q1356) up to 15.4% (between *T*. *apogeana* and *Theileria* sp. B60) (S3 Table).

Genetic distances between representatives of novel sub-clades revealed by the 18S reconstructions were significantly greater at the *cox 3* gene compared to the 18S locus. For instance, genetic distances between quokka-derived genotypes (e.g. Q1356) and isolate TP13 were approximately 10% (S3 Table), whereas at the 18S locus, these distances varied only up to 3.1%.

Novel *cox3* DNA sequences were also obtained from *B*. *lohae*, *Babesia* sp. BP7 and *Babesia* sp. BP1 isolated from Australian wildlife (Fig 2) and represent the first molecular characterisation of Australian *Babesia* spp. at this locus. These sequences clustered together in a well-supported clade within the *Babesia* sensu stricto group and shared from 92.5% (*B*. *lohae* vs *Babesia* sp. BP1) up to 99.2% (*B*. *lohae* vs *Babesia* sp. BP7) genetic identity amongst each other. When compared to other representatives of the *Babesia* sensu stricto group, these genotypes were genetically distinct but most similar to *Babesia* sp. Coco (with genetic distances ranging from 21.3–23.1%) and *B*. *bigemina* (with genetic distances ranging from 22.2–23.4%) (S3 Table). In a similar manner, *cytB* sequences amplified from *Babesia* sp. BP7 and *Babesia* sp. BP1 also formed a well-supported clade within the *Babesia* sensu stricto group (Fig 3). These two genotypes shared 94.7% genetic identity to each other and were most closely related to *Babesia* sp. Coco (genetic distances = 22.5–23.1%) and *B*. *caballi* (genetic distances = 23.1–24.1%) (S4 Table).

Phylogenetic analyses at the *cytB* gene also supported the uniqueness of *Theileria* spp. of Australian marsupials and ticks in the context of other well recognised lineages of piroplasms for which *cytB* sequences are currently available. This novel taxonomic group comprising genotypes and species from Australian marsupials and ticks was most closely related to *Cytauxzoon felis* (Fig 3), with a relatively large genetic distance (21.5%) (S4 Table). Genetic distances within the Australian clade were also significantly greater than those observed at the 18S (S1 Table), and varied from 0.8% (between Q2031b and Q1356) up to 16.1% (between *T*. *apogeana* and TB105) (S4 Table). Importantly, analysis at the *cytB* gene corroborated the existence of a novel *Theileria* genotype within sample TB105, which was genetically distinct but most closely related to *Theileria* sp. B60 (genetic distance = 13.1%) and *T*. *paparinii* (genetic distance = 13.5%) (S4 Table).

The fact that potoroo and quokka-derived samples harboured co-infections with *T*. *worthingtonorum* and *T*. *gilberti* or *T*. *brachyuri*, respectively, associated with a yet incomplete database of mitochondrial gene information from many *Theileria* spp., hinders an accurate and unambiguous characterisation of these isolates at both *cox3* and *cytB* loci (Figs 2 and 3).

### Prevalence, host diversity and spatial distribution of Australian piroplasms

**Fig 4:**
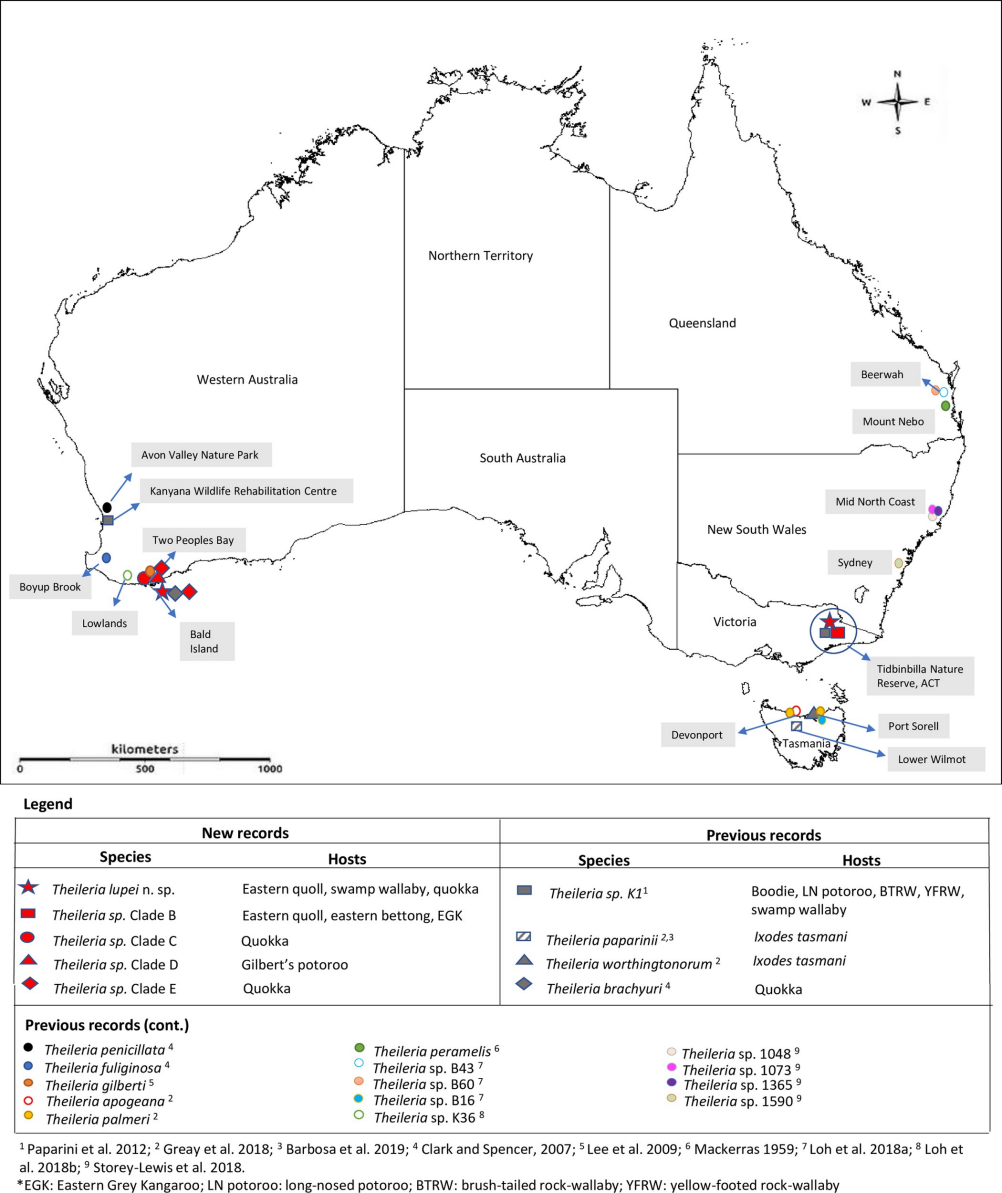
Approximate location (where known) of *Theileria* spp. from marsupials and ticks recorded in Australia to date. Icons in red represent the spatial distribution and host-range of novel *Theileria* spp. identified in the present study.

Molecular analyses revealed the presence of *Theileria* spp. in 100% of the samples tested. A unique *Theileria* sp. (clade A) was detected in an eastern quoll and a swamp wallaby, both from Tidbinbilla Nature Reserve, ACT; and in a quokka from Bald Island in WA. Novel *Theileria* genotypes (clade B) most closely related to *T*. *paparinii* were detected in an eastern bettong, eastern quoll and eastern grey kangaroo, all from Tidbinbilla Nature Reserve. Additionally, a novel genotype (clade E) genetically closely related to *T*. *worthingtonorum* was also identified in 22 quokkas out of 24 (91.6%), of which 17 were from Two Peoples Bay and the remaining five from Bald Island. Of these, four quokkas from Two Peoples Bay, WA, were co-infected with *T*. *brachyury*. Both of the Gilbert’s potoroo samples analysed in the present study had co-infections with *T*. *gilberti* and potential genetic variants of *T*. *worthingtonorum*. The extended spatial distribution and host-range of *Theileria* spp. from marsupials and ticks in Australia are presented in Fig 4.

### Microscopic detection of Australian piroplasms

**Fig 5:**
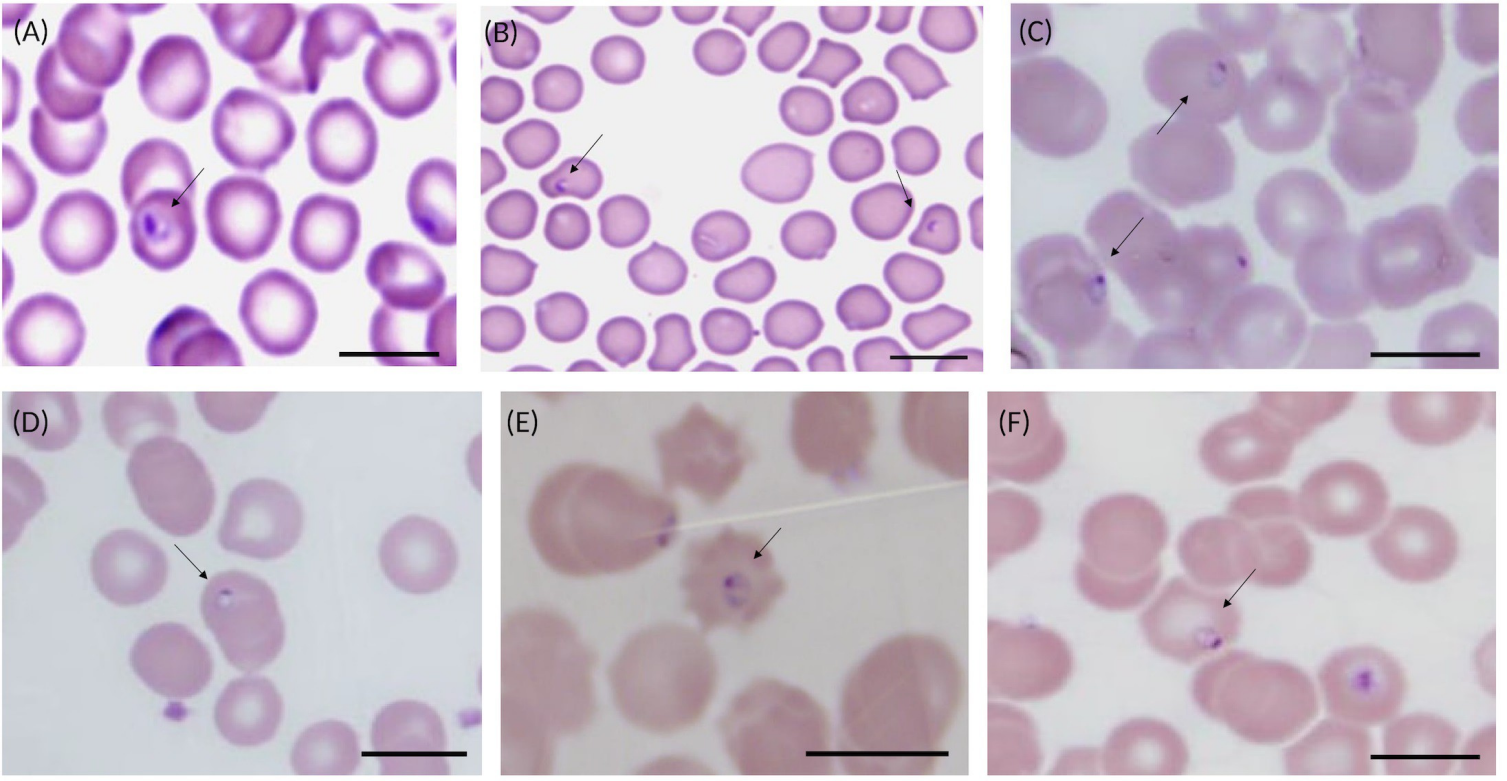
Microscopic detection of intra-erythrocytic ring-shaped piroplasms in blood films from. **(A) eastern quoll (*Dasyurus viverrinus*) (TB105); (B) eastern bettong (*Bettongia gaimardi*) (TB27); (C) quokka (*Setonix brachyurus*) (Q1051); (D) quokka (Q2031a); (E) Gilbert’s potoroo (*Potorous gilbertii*) (P92); (F) quokka (Q1416).** Scale bars represent 10 μm.

Intra-erythrocytic ring-shaped organisms morphologically consistent with piroplasm trophozoites were detected by light microscopy in blood films from six marsupials which were PCR positive for each novel *Theileria* sp. revealed by phylogenetic analysis. The organisms ranged from 1 to 1.7 μm in diameter and presented a darkly basophilic nuclear material and pale basophilic cytoplasm with a fine limiting membrane (Fig 5). Piroplasms observed within the erythrocyte of an eastern quoll (Fig 5A) most likely represent the unique *Theileria* sp. from clade A (Fig 1) and TB105 (Fig 3), since there was no molecular evidence of co-infections in this sample. Intra-erythrocytic organisms morphologically consistent with piroplasms were observed in the blood of an eastern bettong (TB27) (Fig 5B) and may likely represent the same *Theileria* spp. (clade B) identified in this vertebrate host by molecular analysis.

Piroplasmid parasites observed in blood films from quokkas (Q1051 and Q2031a) and a potoroo (P92) (Fig 5C–5E) could not be assigned to a species based on light microscopy only, since these animals had co-infections with *Theileria* spp. belonging to clades C and D, respectively; and *T*. *brachyuri* or *T*. *gilberti*, respectively. Conversely, since there was no molecular evidence of mixed infections in quokka Q1416 (Clade E—Fig 1), the ring-shaped trophozoites visualised in the erythrocytes of this animal may constitute morphological evidence for the novel quokka-derived *Theileria* genotype (clade E) (Fig 5F). No evidence of extra-erythrocytic schizogony was observed in any of the blood smears analysed in the present study.

### Description of novel *Theileria* species

Consistent molecular, phylogenetic and morphological evidence was provided in the present study for a novel *Theileria* species, for which we propose the name *Theileria lupei* n. sp. (Apicomplexa: Theileriidae), in three native marsupial species (eastern quoll, swamp wallaby and quokka) from two different locations in Australia (ACT and Bald Island, WA).

### *Theileria lupei* n. sp.

#### Type-host

Eastern quoll (*Dasyurus viverrinus*)

#### Other hosts

Swamp wallaby (*Wallabia bicolor*); Quokka (*Setonix brachyurus*)

#### Type-locality

Tidbinbilla Nature Reserve (35°27′47″S 148°54′48″E), Australian Capital Territory.

#### Other localities

Bald Island (-34°55'5.43" S 118°27'27.28" E), Western Australia.

#### Type-material

Genomic DNA and a stained thin blood smear from an eastern quoll containing the holotype (Fig 5A) have been deposited at the Western Australian Museum under the accession number WAM Z99098.

#### Representative DNA sequences

DNA sequences were deposited in GenBank under the accessions MN101137 (1,494 bp 18S rRNA gene) and MN135565 (1,072 bp *CytB* gene).

#### Vector

Unknown.

#### ZooBank registration

To comply with the regulations set out in article 8.5 of the amended 2012 version of the International Code of Zoological Nomenclature (ICZN), details of the new species have been submitted to ZooBank. The LSID for the new name *Theileria lupei* is urn:lsid:zoobank.org:act:C6CA31A3-85A7-46ED-9B75-0C96A230A2FA.

#### Etymology

The species is named after Professor Guadalupe Miró Corrales, from the Facultad de Veterinaria, Universidad Complutense de Madrid, in recognition of her enduring contributions to the field of veterinary parasitology.

### Diagnosis

At the 18S locus, the novel species formed a separate clade with strong statistical support (99%) within the group comprising piroplasms from Australian marsupials and ticks (Fig 1A). *Theileria lupei* n. sp. exhibited 2.8% genetic distance from its most closely related organism (*T*. *paparinii*), which was greater than the minimum distance (0.3%) between two previously named piroplasm species (*T*. *annulata and T*. *lestoquardi*), and the distance between many other known species (e.g. *T*. *apogeana* vs *T*. *paparinii*—GD = 1.9%) (S1 Table). In addition, phylogenetic and genetic distance analyses based on a shorter alignment (~600 bp) (Fig 1; S1 and S2 Tables) confirm *T*. *lupei* n. sp. differs from all known theilerias recorded to date, including *T*. *gilberti*, *T*. *brachyuri*, *T*. *fuliginosa*, *T*. *penicillata*, for which only relatively shorter sequences were available. Phylogenetic analysis at the *cytB* locus confirmed the uniqueness of *T*. *lupei* n. sp. compared to other previously recorded theilerias from Australian mammals and ticks. The novel *Theileria* sp. was genetically distinct but most closely related to *Theileria* sp. B60 (genetic distance = 13.1%) and *T*. *paparinii* (genetic distance = 13.5%). These genetic distances were greater than the minimum distance between two distinct species in the analysis (e.g. *T*. *apogeana* and *T*. *paparinii*—genetic distance = 11.9%).

The presence of *T*. *lupei* n. sp. was also confirmed by light microscopy in the blood of an eastern quoll. The parasites presented as small single intra-erythrocytic ring-shaped trophozoites, measuring 1 to 1.7 μm in diameter, with a dark nuclear material and pale basophilic cytoplasm with a fine limiting membrane (Fig 5A). As previously reported, morphological characters alone do not consist an appropriate tool to differentiate between many species of piroplasms as these exhibit highly similar morphometry [3, 20, 26]. Thus, despite clear genetic distinctiveness from other theilerias demonstrated using two genetic markers, the morphometry of *T*. *lupei* n. sp. was indistinguishable from that of other known *Theileria* spp. from Australian marsupials.

## Discussion

The present study describes a novel *Theileria* sp. from Australian marsupials and advances the knowledge of molecular phylogeny of the order Piroplasmida, based on increased taxa sample array and utilisation of multiple genetic markers for molecular characterisation. In particular, this research presents an improved phylogenetic description of *Theileria* spp. from Australian marsupials and ticks, and reports novel epidemiological data such as extended host-ranges and geographic distribution of these parasites.

At the time the first set of *Theileria* 18S sequences was obtained from Australian native marsupials over a decade ago [26], no extra-erythrocytic schizonts (also referred to as Koch’s bodies, a distinguishing feature of the *Theileria* spp.) were observed, and the morphology-based identifications were only possible at order level (i.e. Piroplasmida sp.). Therefore, only molecular data using relatively short sequences were used to describe three (morphologically indistinguishable) novel species: *T*. *brachyuri*, *T*. *fuliginosa*, and *T*. *penicillata* [26]. The tree presented by Clark and Spencer in 2007 [26] clearly shows the novelty of the species, but includes only theilerids, no true babesias, and uses the *Theileria* sp. sensu lato *T*. *bicornis* as outgroup. As a consequence, *T*. *brachyuri*, *T*. *fuliginosa*, and *T*. *penicillata* appeared more similar to *T*. *bicornis* than to the group of true theilerias [26], however, *T*. *bicornis* cannot be confirmed as a *Theileria* sp. because schizonts have never been observed [16]. Following that study, more Australian putative theilerias from marsupials were identified and have been classified taxonomically based on partial DNA fragments [3, 4, 20].

More recent studies have generated nearly complete 18S sequences from some Australian babesias and theilerias, and have shown that phylogenetic analysis of piroplasms based on relatively short fragments (~600–800 bp) generates trees with inconsistencies and features which are resolved only by further analyses of full-length sequences [1, 6, 7, 15, 19, 28]. Here we further characterise previously reported Australian piroplasms and describe novel isolates based on nearly complete 18S sequences. Furthermore, this study reports the first molecular characterisation of Australian marsupial and tick-derived *Babesia* sp. and *Theileria* sp. at the *cox3* and *cytB* loci, thus providing a greater understanding of their phylogeny and genetic diversity.

Phylogenetic analysis at the 18S locus corroborates the position of Australian (putative) *Theileria* spp. outside of the clades containing the equine theilerias (i.e., *T*. *equi*), the true theilerids [7, 15, 20], and the more ancestral theilerias from Australian monotremes [19, 21]. Additionally, novel phylogenetic reconstructions at two mitochondrial genes (*cox3* and *cytB*), which included for the first time sequences from Australian piroplasms (obtained in the present study), provided supporting evidence for the position and uniqueness of this group. Collectively, these results cast doubts on the taxonomic identity of Australian putative theilerias, and prompt the hypothesis that these organisms could in fact represent another genus. Moreover, the fact that no study to date (including the present one), has found evidence of extra-erythrocytic schizogony in blood films from Australian marsupials positive for *Theileria* sp. DNA (even though extra-erythrocytic schizonts are usually difficult to detect [8]), also challenges the assignation of all known marsupial theilerias to the genus *Theileria*. Although intriguing, these observations, combined with the interleaving of other known groups of putative *Theileria* and *Babesia* spp., unresolved clades, soft polytomies, misnomers and underrepresentation of sequences from wildlife, reinforce the necessity of a thorough revision of the systematics of the Piroplasmida as previously suggested (e.g. [1, 8, 15, 19, 20]). In terms of evolutionary relationships, the identification of additional novel *Theileria* spp. presumably unique to Australian marsupials associated with the hitherto absence of other Piroplasmida lineages in these hosts, supports the hypothesis that diversification of most piroplasmids corresponds with the evolution of their mammalian hosts, suggesting co-speciation as a major evolutionary driving force [15].

As expected, because mitochondrial genomes evolve more rapidly than conserved non-coding nuclear genes [35], pairwise distances between *cox3* and *cytB* sequences were significantly greater compared to the distances between corresponding pairs of 18S sequences. This new data sheds more light onto the remarkable intra- and inter-specific genetic diversity of Australian marsupial-derived piroplasms revealed by the 18S analysis, since most of these organisms, although phylogenetically distinct, are highly similar (>97%) to each other at this gene. In cases where mitochondrial genome or whole genome sequencing are not feasible, the authors recommend that molecular assays targeting mitochondrial genes continue to be used in association with 18S analysis for future research aiming to identify and characterise Australian piroplasms. As more genetic information at the *cox3* and *cytb* genes become available from representative species and within-species genetic variants, we anticipate that improved phylogenetic resolution will be achieved and therefore existing lineages will be further refined.

Phylogenetic reconstructions and estimates of genetic distances at the 18S and *cytB* genes provided compelling evidence of a novel piroplasm species from Australian marsupials, for which we have proposed the name *Theileria lupei* n. sp. This discovery was also supported by visual identification of the parasite by light microscopy, morphometric description and the amplification of nearly identical *T*. *lupei* n. sp. 18S sequences from three distinct hosts (an eastern quoll, a swamp wallaby and a quokka) from two different locations (Tidbinbilla Reserve, ACT and Bald Island, WA). The formal species description presented here complies with the *International Code of Zoological Nomenclature* and takes into consideration important taxonomic requirements recently debated in the literature [14, 36]. We agree that even though the 18S locus is the most widely used locus to delimit haemoparasite species, description of additional genetic markers and morphological features aids the phylogenetic resolution of these organisms and offers a more complete understanding of the characters of a species compared to a single genetic marker [14, 37, 38]. As with any novel species, the discovery of *T*. *lupei* n. sp. from Australian marsupials prompts the necessity of further investigation to determine its full host-range, vectors, potential pathogenicity and zoonotic potential.

Phylogenetic analyses and estimates of evolutionary divergence based on nearly complete 18S sequences suggest that novel *Theileria* spp. to clades B, C and E could potentially represent distinct species from their most closely related named taxa. However, further research is required to confirm this hypothesis. Evolutionary analyses also revealed an increased genetic diversity within the clade containing the previously described *T*. *paparinii* and *Theileria* sp. K1 [7, 20], which now also encompasses novel genotypes designated as sub-clades B and C. Furthermore, in addition to the boodie, brush-tailed rock-wallaby (*P*. *penicillata*), yellow-footed rock-wallaby (*P*. *xanthopus*) and swamp wallaby [5, 20], the host range associated with the *T*. *paparinii* clade, now includes the eastern quoll and eastern bettong. Since there are no long (~1.4kb) 18S fragments available for *T*. *penicillata* from the woylie [26] and *Theileria* sp. from the long-nosed potoroo [3], the taxonomic identity of these organisms in the context of nearly full-length 18S sequence analysis could not be ascertained.

Phylogenetic reconstructions suggest that long *Theileria* sp. 18S fragments from samples Q2031a and Q1051 (clade C) or *Theileria* spp. belonging to clade E might correspond to the previously described *T*. *brachyuri* [26]. However, analyses of type-specimens associated with *T*. *brachyuri* (not available in the present study) are required in order to confirm this assumption. Interestingly, both of the Gilbert’s potoroo blood samples tested in this study harboured both *T*. *gilberti* and *Theileria* sp. (clade D). Further research is required to determine the species status of clade D as although the genetic distance between quokka-derived representatives of this clade and *T*. *worthingtonorum* was relatively short (only 0.1%) at the 18S locus, this is a slow-evolving gene and not surprisingly, a few distinct species of the Apicomplexa have been found to be identical at this locus [39].

Our results discussed above constitute novel evidence that mixed piroplasm infections may be common in Australian marsupials, particularly in quokkas and potoroos. Co-infections are recognised as a conservation challenge, as synergistic interaction effects among multiple pathogens may alter their collective pathogenicity [40]. Thus, since clean Sanger sequencing chromatograms (as those obtained in the present study) do not rule out the presence of concurrent infections [41], alternative tools such as species-specific PCRs or, preferably, targeted amplicon next-generation sequencing (NGS) are required to simultaneously characterise co-infecting species and investigate their potential impact on Australian marsupials.

From an epidemiological perspective, although *T*. *worthingtonorum* has been identified in *I*. *tasmani* ticks, it is not known whether this tick species is a competent vector or whether this piroplasm species is infective to dogs. However, the potential finding of *T*. *worthingtonorum* in wildlife species suggests that these could be the main reservoir hosts of these parasites, and the original blood meal source of the tick infections reported by Greay et al. [7]. From a One Health viewpoint, the lack of host-specificity of *I*. *tasmani* [42] leads to the speculation that this tick could potentially transmit *T*. *worthingtonorum* from wildlife to dogs, livestock and people. Further research on the life-cycle, hosts and potential pathogenicity of *T*. *worthingtonorum* are required to confirm this hypothesis and assess any potential veterinary or medical impacts. Furthermore, even though no exotic pathogens were detected in the present study, ongoing surveillance is recommended within the current paradigm of increased anthropogenically-mediated animal movement globally, where wildlife reservoirs of infectious agents potentially pose a risk of spread of new diseases to domesticated animals and people [43].

This study also reports the first molecular characterisation of Australian *Babesia* spp. *(B*. *lohae*, and *Babesia* sp. BP1) at the *cox3* locus. As in the 18S analysis, these organisms clustered within the *Babesia* sensu lato clade, which contains many pathogens of veterinary importance, including *B*. *macropus* from the eastern grey kangaroo [27, 44]. The epidemiology and potential pathogenicity of *B*. *lohae*, and *Babesia* sp. BP1 warrants further investigation.

Microscopy analysis confirmed the presence of these parasites in at least one marsupial host, positive for each of the novel genotypes detected by PCR. The size range of the organisms detected was within the range typically observed in Australian marsupial theilerias (0.4–3.1 μm) [3, 20, 26, 45]. This corroborates previous observations that morphometry of small theilerias from Australian wildlife are very similar, and thus these organisms are morphologically indistinguishable.

## Conclusions

This study further characterised *Theileria* spp. from Australian marsupials and ticks using microscopy and multiple genetic markers, the combination of which has led to the discovery of a novel *Theileria* sp. (named *T*. *lupei* n. sp.). In addition, the findings support the polyphyly and ubiquitousness of the genus *Theileria* as well as an increased host-range and inter and intra-specific genetic diversity within the clade comprising Australian marsupial-derived theilerias. This research also draws attention to the fact that mixed piroplasm infection may be common in wildlife, thus the development of a NGS-based assay specifically targeting piroplasms is required.

Importantly, the present study constitutes a new effort to better understand the overall systematics of Piroplasmida and therefore represents another step towards a taxonomic re-classification of the order. In order to achieve that, future research should focus on establishing more well-defined taxonomic boundaries within the Piroplasmida through: more extensive sampling; elucidation of life-cycles and potential impact of presumably non-pathogenic species; use of mitochondrial genetic markers; and revision of outdated classification criteria (e.g. morphology-based only identifications and biological traits known to be common to different taxa).

## Supporting information

S1 Table Estimates of evolutionary divergence between piroplasm 18S rDNA sequences (~1.3 kb). Pairwise genetic distances matrix was obtained using the General Time Reversible model (Nei and Kumar, 2000). Duplicates were removed from the database therefore only one representative of each novel sequence/clade is shown.
(PDF)

S2 Table Pairwise genetic distances matrix showing the genetic diversity among *Theileria* spp. from Australian marsupials and ticks at the 18S rRNA locus. Analyses were conducted on a ~ 800 bp alignment using the Tamura-Nei model (Tamura and Nei, 1993). Duplicates were removed from the database therefore only one representative of each novel sequence/clade is shown. * indicates shorter sequences (~800 bp) obtained in this study using a nested PCR as described by Jefferies et. al. (2007). All other new sequences (~1.3 kb) were obtained using primers Nbab_1F and 18S ApiR (Greay et al., 2018).
(PDF)

S3 Table Estimates of evolutionary divergence between piroplasm *cox3* nucleotide sequences (~ 600 bp). Pairwise genetic distances (%) obtained using the p-distance method are shown.
(PDF)

S4 Table Estimates of evolutionary divergence between piroplasm *cytB* nucleotide sequences (~ 1.1 kb). Pairwise genetic distances (%) obtained using the p-distance method are shown.
(PDF)
