# Supplementary material for: Sequence analyses at mitochondrial and nuclear loci reveal a novel Theileria sp. and aid in the phylogenetic resolution of piroplasms from Australian marsupials and ticks
Source: PLoS One. 2019 Dec 18;14(12):e0225822. doi: 10.1371/journal.pone.0225822 (PMC6919580; doi:10.1371/journal.pone.0225822)
Supplement: S2 Table — Analyses were conducted on a ~ 800 bp alignment using the Tamura-Nei model (Tamura and Nei, 1993). Duplicates were removed from the database therefore only one representative of each novel sequence/clade is shown. * indicates shorter sequences (~800 bp) obtained in this study using a nested PCR as described by Jefferies et. al. (2007). All other new sequences (~1.3 kb) were obtained using primers Nbab_1F and 18S ApiR (Greay et al., 2018). (PDF) [file pone.0225822.s002.pdf]

	TP13 - CLADE B	TP8 - CLADE B	*Theileria* sp.(EF554395)	*Theileria paparinii* (MG758115)	*Theileria penicillata* (DQ437687)	*Theileria* sp. K1 (JQ682879)	**Q2031b* - *T. brachyuri* CLADE**	**Q1340* - *T. brachyuri* CLADE**	**1051 - *T. brachyuri* CLADE**	**P92* and P68* - *T. gilberti* CLADE**	*Theileria gilberti* (EF554394)	**Q2031a - *T. brachyuri* CLADE**	*Theileria brachyuri* (DQ437684)	*Theileria* sp. B16 (MG251437)	*Theileria* sp. B60 (MG251439)	*Theileria* sp. B43 (MG251438)	*Theileria apogeana* (MG758116)	**Q2031b - CLADE E**	*Theileria worthingtonorum* (MG758114)	**P92 and P68 - CLADE D**	*Theileria* sp. (MF576261)	*Theileria* sp. (MF136488)	*Theileria palmeri* (MG758113)	**TB111 - CLADE A**	**TB105 - CLADE A**	**Q2377 - CLADE A**	*Theileria* sp. (MF136494)	*Theileria fuliginosa* (DQ437686)	*Theileria* sp. (MF136493)
**TP13 - CLADE B**																													
**TP8 - CLADE B**	0.2%																												
Theileria sp.(EF554395)	0.2%	0.0%																											
Theileria paparinii (MG758115)	0.2%	0.0%	0.0%																										
Theileria penicillata (DQ437687)	0.6%	0.4%	0.4%	0.4%																									
Theileria sp. K1 (JQ682879)	0.8%	0.6%	0.6%	0.6%	0.2%																								
**Q2031b* - T. brachyuri CLADE**	1.7%	1.4%	1.4%	1.4%	1.4%	1.2%																							
**Q1340* - *T. brachyuri* CLADE**	1.7%	1.4%	1.4%	1.4%	1.4%	1.2%	0.0%																						
**1051 - T. brachyuri CLADE**	1.7%	1.4%	1.4%	1.4%	1.4%	1.2%	0.0%	0.0%																					
**P92* and P68* - *T. gilberti* CLADE**	2.5%	2.2%	2.2%	2.2%	2.2%	1.9%	0.6%	0.6%	0.6%																				
Theileria gilberti (EF554394)	2.7%	2.4%	2.4%	2.4%	2.4%	2.1%	0.7%	0.7%	0.7%	0.2%																			
**Q2031a - *T. brachyuri* CLADE**	1.7%	1.4%	1.4%	1.4%	1.4%	1.2%	0.0%	0.0%	0.0%	0.6%	0.7%																		
Theileria brachyuri (DQ437684)	1.4%	1.2%	1.2%	1.2%	1.7%	1.4%	0.2%	0.2%	0.2%	0.8%	1.0%	0.2%																	
Theileria sp. B16 (MG251437)	3.4%	3.0%	3.0%	3.0%	3.0%	2.7%	3.8%	3.8%	3.8%	4.7%	4.9%	3.8%	4.2%																
Theileria sp. B60 (MG251439)	3.4%	3.0%	3.0%	3.0%	3.0%	2.7%	3.8%	3.8%	3.8%	4.7%	4.9%	3.8%	4.2%	0.0%															
Theileria sp. B43 (MG251438)	4.0%	3.6%	3.6%	3.6%	3.6%	3.2%	3.6%	3.6%	3.6%	4.7%	4.9%	3.6%	4.0%	1.4%	1.4%														
Theileria apogeana (MG758116)	3.4%	3.0%	3.0%	3.0%	3.7%	3.4%	4.1%	4.1%	4.1%	4.3%	4.6%	4.1%	3.7%	5.9%	5.9%	5.0%													
**Q2031b - CLADE E**	4.6%	4.2%	4.2%	4.2%	5.1%	5.6%	5.4%	5.4%	5.4%	4.6%	4.8%	5.4%	4.9%	7.9%	7.9%	6.7%	4.1%												
Theileria worthingtonorum (MG758114)	5.5%	5.1%	5.1%	5.1%	6.0%	6.5%	6.2%	6.2%	6.2%	5.3%	5.5%	6.2%	5.7%	7.2%	7.2%	6.6%	4.6%	1.4%											
**P92 and P68 - CLADE D**	4.9%	4.4%	4.4%	4.4%	5.3%	5.8%	5.5%	5.5%	5.5%	4.9%	5.1%	5.5%	5.0%	8.0%	8.0%	6.9%	4.0%	0.9%	0.4%										
Theileria sp. (MF576261)	6.8%	6.3%	6.3%	6.3%	7.3%	7.9%	7.9%	7.9%	7.9%	7.3%	7.6%	7.9%	7.3%	10.0%	10.0%	10.8%	7.9%	4.2%	2.3%	2.8%									
Theileria sp. (MF136488)	6.3%	5.8%	5.8%	5.8%	5.8%	6.3%	4.8%	4.8%	4.8%	4.2%	4.4%	4.8%	5.3%	5.5%	5.5%	5.2%	9.0%	6.1%	6.6%	5.9%	8.2%								
Theileria palmeri (MG758113)	10.4%	9.6%	9.6%	9.6%	8.3%	8.9%	8.2%	8.2%	8.2%	7.1%	7.4%	8.2%	8.8%	10.0%	10.0%	10.8%	12.0%	7.1%	7.2%	8.0%	6.0%	7.4%							
**TB111 - CLADE A**	8.3%	7.8%	7.8%	7.8%	6.8%	6.4%	5.8%	5.8%	5.8%	5.5%	5.7%	5.8%	6.3%	9.0%	9.0%	9.3%	9.3%	8.0%	8.1%	8.3%	7.6%	6.7%	7.3%						
**TB105 - CLADE A**	8.3%	7.8%	7.8%	7.8%	6.8%	6.4%	5.8%	5.8%	5.8%	5.5%	5.7%	5.8%	6.3%	9.0%	9.0%	9.3%	9.3%	8.0%	8.1%	8.3%	7.6%	6.7%	7.3%	0.0%					
**Q2377 - CLADE A**	8.3%	7.8%	7.8%	7.8%	6.9%	6.4%	5.9%	5.9%	5.9%	5.9%	6.1%	5.9%	6.3%	9.0%	9.0%	9.3%	9.9%	8.6%	8.7%	8.9%	8.0%	7.2%	7.8%	0.4%	0.4%				
Theileria sp. (MF136494)	5.9%	5.4%	5.4%	5.4%	5.4%	5.0%	3.4%	3.4%	3.4%	3.6%	3.9%	3.4%	3.7%	6.1%	6.1%	6.5%	7.8%	7.9%	9.6%	8.7%	12.4%	4.8%	9.9%	7.8%	7.8%	8.3%			
Theileria fuliginosa (DQ437686)	9.0%	8.5%	8.5%	8.5%	9.6%	10.2%	10.2%	10.2%	10.2%	9.6%	9.9%	10.2%	9.5%	12.6%	12.6%	13.5%	10.2%	6.0%	4.2%	4.8%	1.5%	10.6%	8.1%	10.0%	10.0%	10.4%	15.3%		
Theileria sp. (MF136493)	14.0%	13.1%	13.1%	13.1%	11.5%	10.8%	11.2%	11.2%	11.2%	9.8%	10.1%	11.2%	11.9%	10.6%	10.6%	10.2%	12.6%	17.5%	14.6%	15.6%	18.6%	10.4%	12.3%	12.1%	12.1%	12.9%	10.0%	21.4%	
