# Supplementary material for: Sequence analyses at mitochondrial and nuclear loci reveal a novel Theileria sp. and aid in the phylogenetic resolution of piroplasms from Australian marsupials and ticks
Source: PLoS One. 2019 Dec 18;14(12):e0225822. doi: 10.1371/journal.pone.0225822 (PMC6919580; doi:10.1371/journal.pone.0225822)
Supplement: S3 Table — Pairwise genetic distances (%) obtained using the p-distance method are shown. (PDF) [file pone.0225822.s003.pdf]

	**Q2367**	**Q2031b**	**Q1349**	**Q1356**	**TP13**	***Theileria* sp. B16**	***Theileria* sp. B60**	***Theileria paparinii***	**P92**	**P68**	***Theileria apogeana***	***Babesia* sp. BP1**	***Babesia* sp. B7**	***Babesia lohae***	***Babesia gibsoni***	*Babesia gibsoni* (AB499087)	*Babesia canis canis* (KC207822)	*Babesia canis vogeli* (KC207825)	*Babesia canis rossi* (KC207823)	*Babesia caballi* (AB499086)	*Babesia* sp. Coco (KC207824)	*Babesia bigemina* (AB499085)	*Babesia bovis* (AB499088)	*Theileria annulata* (NW 001091933)	*Theileria parva* (AB499089)	*Cytauxzoon felis* (KC207821)	*Theileria orientalis* (AB499090)	*Babesia conradae* (KC207826)	*Babesia microti* (LN871600)	*Babesia microti* (AB624353)	*Babesia cf. microti* (AB624353)	*Babesia rodhaini* (AB624357)	*Theileria equi* (AB499091)	*Plasmodium falcipar*
**Q2367**																																		
**Q2031b**	0.8%																																	
**Q1349**	0.8%	0.0%																																
**Q1356**	1.5%	0.6%	0.6%																															
**TP13**	9.6%	9.9%	9.9%	9.6%																														
***Theileria* sp. B16**	13.3%	12.8%	12.8%	12.2%	12.5%																													
***Theileria* sp. B60**	13.8%	13.3%	13.3%	12.7%	13.1%	2.4%																												
Theileria paparinii	13.5%	13.5%	13.5%	12.8%	12.3%	11.5%	11.4%																											
**P92**	12.3%	12.0%	12.0%	11.7%	10.6%	14.9%	14.9%	14.1%																										
**P68**	12.3%	12.0%	12.0%	11.7%	10.6%	14.9%	14.9%	14.1%	0.0%																									
Theileria apogeana	12.5%	12.2%	12.2%	11.9%	11.5%	14.9%	15.4%	13.8%	3.9%	3.9%																								
***Babesia* sp. BP1**	40.3%	39.9%	39.9%	39.4%	38.5%	39.6%	39.8%	38.8%	37.8%	37.8%	38.1%																							
***Babesia* sp. B7**	39.9%	39.6%	39.6%	39.1%	38.3%	38.6%	38.8%	38.3%	37.0%	37.0%	37.5%	7.3%																						
Babesia lohae	39.9%	39.6%	39.6%	39.1%	37.8%	38.3%	38.5%	38.1%	36.9%	36.9%	37.3%	7.5%	0.8%																					
Babesia gibsoni	36.5%	36.4%	36.4%	36.0%	34.4%	33.9%	33.8%	35.9%	35.1%	35.1%	35.1%	29.9%	29.1%	29.2%																				
Babesia gibsoni (AB499087)	36.5%	36.4%	36.4%	36.0%	34.4%	33.9%	33.8%	35.9%	35.1%	35.1%	35.1%	29.9%	29.1%	29.2%	0.0%																			
Babesia canis canis (KC207822)	37.7%	37.5%	37.5%	37.2%	36.7%	34.4%	34.7%	36.9%	36.5%	36.5%	35.2%	29.2%	27.1%	27.3%	15.7%	15.7%																		
Babesia canis vogeli (KC207825)	39.9%	39.8%	39.8%	39.4%	39.8%	39.0%	39.1%	40.3%	39.0%	39.0%	38.1%	32.6%	31.2%	31.3%	22.6%	22.6%	16.1%																	
Babesia canis rossi (KC207823)	41.1%	40.7%	40.7%	40.4%	40.3%	37.2%	37.5%	41.6%	40.4%	40.4%	40.9%	32.8%	31.2%	31.0%	23.1%	23.1%	21.1%	25.8%																
Babesia caballi (AB499086)	38.0%	38.0%	38.0%	37.7%	36.5%	35.7%	36.0%	36.5%	36.9%	36.9%	37.7%	23.7%	22.9%	23.2%	26.9%	26.9%	24.4%	29.7%	30.5%															
Babesia sp. Coco (KC207824)	39.9%	39.6%	39.6%	39.1%	38.8%	36.7%	36.7%	39.0%	38.6%	38.6%	38.8%	23.1%	21.3%	21.3%	27.4%	27.4%	26.3%	31.3%	28.2%	19.2%														
Babesia bigemina (AB499085)	40.4%	40.4%	40.4%	39.9%	38.3%	37.8%	38.6%	40.3%	39.3%	39.3%	39.8%	23.4%	22.2%	22.2%	27.9%	27.9%	26.1%	32.0%	27.9%	22.2%	18.7%													
Babesia bovis (AB499088)	39.0%	39.0%	39.0%	38.5%	37.8%	37.2%	37.8%	38.8%	37.8%	37.8%	37.8%	30.2%	30.0%	29.7%	32.6%	32.6%	31.5%	36.9%	36.4%	27.9%	29.1%	26.1%												
Theileria annulata (NW 001091933)	30.5%	30.0%	30.0%	29.9%	29.5%	28.1%	27.8%	29.2%	30.2%	30.2%	30.2%	41.6%	40.6%	40.9%	40.1%	40.1%	40.1%	44.2%	41.9%	41.9%	40.6%	42.4%	42.4%											
Theileria parva (AB499089)	29.7%	29.5%	29.5%	28.9%	27.3%	26.3%	26.9%	27.4%	28.4%	28.4%	28.9%	42.0%	42.0%	42.2%	40.9%	40.9%	41.6%	44.6%	40.9%	40.7%	42.5%	42.7%	43.2%	19.6%										
Cytauxzoon felis (KC207821)	28.2%	27.6%	27.6%	27.3%	25.8%	28.1%	28.1%	27.6%	26.8%	26.8%	27.1%	39.6%	39.0%	39.1%	38.0%	38.0%	36.9%	41.2%	38.0%	37.7%	37.2%	40.9%	39.3%	29.9%	27.8%									
Theileria orientalis (AB499090)	31.5%	30.8%	30.8%	30.4%	28.7%	27.6%	27.4%	30.0%	31.8%	31.8%	31.8%	42.2%	41.7%	41.7%	40.1%	40.1%	41.1%	44.0%	41.9%	42.4%	42.4%	42.7%	43.8%	24.8%	24.7%	30.5%								
Babesia conradae (KC207826)	55.8%	55.4%	55.4%	55.2%	55.7%	55.7%	55.4%	55.8%	54.7%	54.7%	54.9%	55.4%	54.1%	54.2%	57.3%	57.3%	56.3%	57.1%	56.3%	55.2%	53.9%	59.3%	58.4%	57.0%	55.4%	55.5%	55.7%							
Babesia microti (LN871600)	52.6%	52.1%	52.1%	52.3%	51.6%	52.8%	53.9%	53.7%	53.6%	53.6%	53.4%	53.2%	53.9%	53.9%	52.8%	52.8%	54.4%	52.3%	52.6%	53.9%	53.2%	55.0%	56.0%	55.7%	54.9%	53.2%	54.9%	63.6%						
Babesia microti (AB624353)	53.6%	53.6%	53.6%	53.7%	52.9%	54.4%	55.7%	54.5%	55.0%	55.0%	54.9%	54.9%	55.4%	55.4%	54.2%	54.2%	55.7%	54.7%	55.8%	54.9%	54.7%	53.9%	55.7%	56.0%	56.7%	53.9%	56.0%	67.0%	16.7%					
Babesia cf. microti (AB624353)	53.1%	53.2%	53.2%	53.1%	51.6%	53.9%	54.7%	53.7%	53.7%	53.7%	52.9%	55.2%	55.2%	55.2%	54.4%	54.4%	55.4%	53.7%	56.3%	56.2%	55.8%	54.4%	56.2%	55.5%	56.0%	54.4%	55.4%	63.0%	18.0%	20.9%				
Babesia rodhaini (AB624357)	53.6%	53.6%	53.6%	53.4%	51.3%	51.3%	51.8%	52.4%	52.1%	52.1%	52.9%	52.8%	52.8%	52.8%	50.8%	50.8%	52.1%	53.4%	55.2%	54.5%	52.3%	54.9%	57.0%	52.9%	53.2%	52.4%	51.1%	62.8%	41.4%	40.6%	39.1%			
Theileria equi (AB499091)	58.6%	58.1%	58.1%	57.9%	58.3%	57.4%	57.9%	58.1%	57.2%	57.2%	57.4%	63.1%	60.7%	60.5%	60.2%	60.2%	59.3%	61.6%	62.1%	60.5%	59.7%	61.4%	61.9%	58.3%	60.0%	58.8%	56.0%	59.8%	62.1%	63.7%	62.1%	60.0%		
Plasmodium falciparum (AY283019)	55.4%	55.1%	55.1%	54.7%	54.4%	52.5%	52.8%	53.2%	54.7%	54.7%	55.3%	58.7%	56.5%	56.1%	57.3%	57.3%	56.1%	58.2%	57.7%	59.6%	58.9%	56.6%	59.4%	56.5%	57.0%	56.3%	57.0%	65.8%	59.9%	57.2%	58.9%	56.8%	61.9%	
